# The influence of body posture on facial expression perception in Autism

**DOI:** 10.1038/s41598-024-79547-0

**Published:** 2024-11-12

**Authors:** Abigail Finn, Punit Shah, Stephan de la Rosa, Christoph Teufel, Elisabeth von dem Hagen

**Affiliations:** 1 Cardiff University Brain Research Imaging Centre, School of Psychology, Cardiff University, Maindy Road, CF24 4HQ Cardiff, UK; 2 Wales Autism Research Centre, School of Psychology, Cardiff University, Cardiff, UK; 3 School of Psychology, University of Bath, Bath, UK; 4 Department of Psychology, IU International University of Applied Sciences, Erfurt, Germany

**Keywords:** Facial expression, Body posture, Context, Emotion, Autism, Autism spectrum disorder, Psychology, Human behaviour

## Abstract

Facial expression perception is influenced by body posture, with perception biased toward the body emotion. Previous research has suggested that the magnitude of this biasing influence of body posture is driven by individual differences in the precision of facial expression representations underlying discrimination abilities, where lower precision leads to a greater influence of body posture. It is unclear however whether similar mechanisms might drive the influence of contextual cues in Autism, which is often characterised by reduced facial expression discrimination abilities. Here, we addressed this question by using online psychophysical methods to determine the precision of isolated face and body expression representations of anger and disgust, and the influence of body on facial expression perception, in autistic and non-autistic adults. Both groups showed a strong influence of body context on facial expression perception, but this influence was larger in the autistic group, mirroring their lower overall precision of facial expression representations relative to non-autistic individuals. Crucially, the magnitude of the biasing influence of body posture in both groups was related to the precision of individuals’ facial expression representations. The results suggest that similar principles govern the integration of facial expression and body posture information in both autistic and non-autistic individuals.

## Introduction

Recognition of other people’s facial expressions is essential for adaptive social behaviour and communication^1^. In everyday life, however, we rarely encounter facial expressions on their own, but typically experience them embedded within a context. One of the most basic but fundamental types of contexts is provided by the other’s body posture. Indeed, a growing literature indicates that the perception of facial expressions can be significantly influenced by body context in neurotypical adults, whereby the perceived expression is biased towards the emotion communicated by the posture^2–4^. For example, a disgusted facial expression is more likely to be perceived as angry when displayed in the context of an angry body posture^2^. This body context effect suggests that emotion perception is based not solely on facial cues but relies on an integration of information from the other person’s face and body, a process that has important implications for how we perceive emotions in real-world settings^5,6^. In the current study, we evaluated the integration of affective face and body cues in individuals with Autism Spectrum Disorders (ASD; henceforth Autism), who are known to have difficulties in social function.

The extent to which body context influences facial expression perception shows large individual differences in adults^4^. It also exhibits a clear developmental trajectory in childhood and adolescence^7^. Specifically, as the ability to discriminate facial expression increases with age, the influence of body posture on facial expression perception decreases, such that younger children show lower facial expression discrimination ability combined with a greater influence of body posture. This behavioural profile is associated with developmental changes in neural microstructure in core face- and body-specific areas, and with changes in the connectivity between face- and body-specific pathways^7^. These behavioural and neurobiological findings point towards a potential link between the reliability with which isolated face signals are represented and the extent to which facial expression perception is influenced by body context. In particular, in line with Bayesian accounts of brain function more generally^8–11^, when integrating facial expressions and body context, the contribution of these cues to the integrated percept might be determined by their relative reliability. To put it simply, according to this perspective, the biasing effect of body context will be stronger in observers who generate less reliable representations of the isolated facial expression compared to those who generate more reliable representations.

This perspective raises important questions regarding emotion perception in developmental disorders such as Autism. Autistic individuals have often been reported to exhibit difficulties in facial expression perception when faces are presented in isolation^12^ (for reviews see:^13–15^ ). Conversely, research suggests that there is no difference in recognition accuracy for emotional body postures in autistic relative to non-autistic individuals^16^. According to the Bayesian perspective, autistic individuals should therefore show a greater influence of body context on facial expression perception compared to neurotypical individuals. However, the only study to date that has investigated the influence of body context on facial expression perception in Autism found no difference in the body context effect between neurotypical and autistic adults^17^. Critically, however, in this study, the two groups also did not differ in their ability to discriminate facial expressions (although there was more variability within the autistic group), a finding that has to be interpreted within the context of a heterogenous association between Autism and poor facial expression recognition. In particular, one of the largest studies to date suggests that different subgroups of autistic individuals show different extents of difficulties in facial expression perception^18^. The autistic individuals in the relatively small sample studied by Brewer and colleagues^17^ might have predominantly come from autistic subgroups with little or no difficulty in facial expression perception. Importantly, from the Bayesian perspective of face-body integration, it is therefore not surprising that these individuals showed similar levels of influence of body posture on facial expression perception as non-autistic individuals.

In the current study, we worked with a much larger sample than the previous study to assess the influence of body posture on facial expression perception in autistic adults, and the extent to which the precision of facial expression representations predicts the influence of body context. Using psychophysical methods, we conducted an online study to investigate the relationship between the precision of isolated facial expression representations and isolated body posture representations, and the influence of body posture on facial expression perception in autistic and neurotypical adults. Across three tasks, participants were asked to discriminate between anger and disgust isolated facial expression morphs (face-only task), body posture morphs (body-only task), and facial expression morphs in the context of an angry or disgusted body posture (face-in-context task). Anger and disgust expressions of face and body were used due to the large influence of body context on facial expression perception with these emotions^2,7^ and to maintain consistency with Brewer et al.^17^. Performance in the three tasks provided indices of the precision of facial expression and body posture representations, and the influence of body posture on facial expression perception, respectively. By contrast to Brewer et al.^17^, we found that autistic individuals showed a larger influence of body posture than non-autistic individuals when making judgments of facial expressions, and this influence of body posture was driven by the precision of facial expression representations in both groups.

## Results

**Fig. 1:**
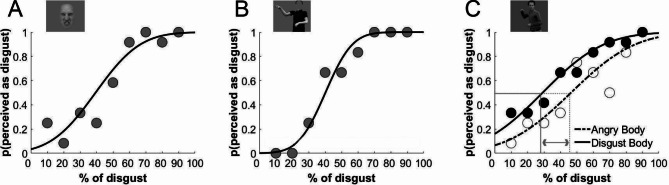
Example psychometric functions from one autistic participant. (**A**) isolated facial expression morphs, (**B**) isolated body posture morphs, (**C**) facial expressions in the context of an angry or disgusted body posture. The estimates for the slope parameter from (A) and (B) are measures of the precision of the representations that underpin discrimination ability, and the difference in PSEs from (C) provides a measure of the influence of body posture on facial expression categorisation (indicated by grey lines and arrow). Each circle represents the average response of 12 trials at a given morph level.

Psychometric functions were fit to each participant’s data from each task, which resulted in 4 functions per participant: one for the task with isolated facial expressions (Fig. 1A), one for the task with isolated body postures (Fig. 1B), and two for the task, in which facial expressions were shown in the context of a body posture (one for facial expressions on an angry body posture, and one for facial expressions on a disgusted body posture (Fig. 1C)).

For the two tasks with isolated cues, we were interested in the estimated values of the slope parameter of the psychometric functions, providing a measure for the precision of facial expression and body posture representations that underpin discrimination ability. Larger slope, or precision, values are reflective of better discrimination ability. For the task in which facial expressions were presented in the context of either an angry or a disgusted body posture, we were interested in the point-of-subjective-equality (PSE) of the psychometric functions. The difference in PSE values between the two psychometric functions provides a measure of the influence of body posture on facial expression perception, since the psychometric functions are based on responses to the same facial expression morphs but in different body contexts. A larger PSE difference between the two functions indicates a larger influence of body posture on facial expression perception.

### Isolated facial expression and isolated body posture perception

**Fig. 2:**
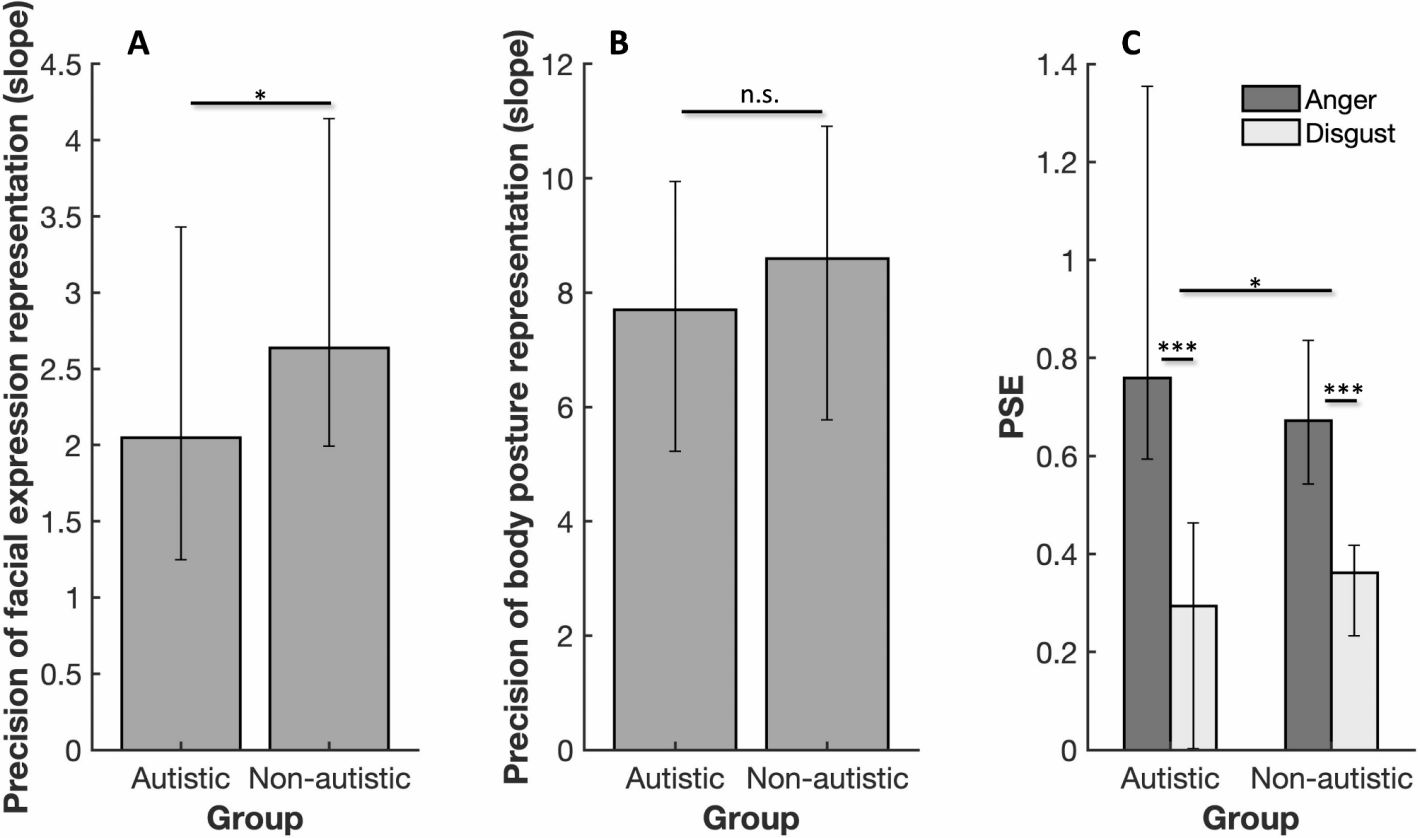
The precision with which isolated facial expressions and isolated body postures are represented in the autistic and non-autistic groups, and the influence of body posture on facial expression perception in both groups. (**A**) Median precision of facial expression representations in the isolated facial expression task in the two groups, as indexed by the estimated slope parameter of the psychometric function. (**B**) Median precision of body posture representations in the isolated body posture task, again indexed by the slope. Error bars in all plots represent the first and the third quartile. (**C**) Median PSE for facial expressions shown on angry body postures (dark grey) and disgusted body postures (light grey). The difference between the dark grey and light grey bars is a measure of the influence of body posture on facial expression perception. Wilcoxon rank sum test p-values: * *p* < 0.05, ** *p* < 0.01, *** *p* < 0.001, n.s. non-significant.

Facial expression discrimination abilities, as measured by the slopes of the psychometric functions in the isolated facial expression task, were significantly lower in the autistic group (median: 2.048; Fig. 2A; Wilcoxon rank sum test z = 2.504, *p* = 0.012) than in the non-autistic group (median: 2.636; Fig. 2A). This suggests that autistic participants had less precise facial expression representations than non-autistic participants. The slopes of the psychometric functions in the isolated body posture task suggested that there was no difference in body posture discrimination abilities between the two groups (Fig. 2B; z = 1.199, *p* = 0.23).

### Influence of body posture on facial expression perception

Prior to assessing the body context effect in both groups, we wanted to establish that participants approached the face-in-context and the face-only tasks in a similar manner, as they were instructed to make judgments about the face only in both tasks. This idea was supported by significant correlations between the slope values in the face-only task and the face-in-context task (Autistic – angry body: Spearman’s rho = 0.51, *p* < 0.001; Autistic – disgusted body: rho = 0.35, *p* = 0.02; Non-Autistic – angry body: rho = 0.38, *p* < 0.005; Non-Autistic – disgusted body: rho = 0.45, *p* < 0.001). In other words, these associations suggest that, similarly to the face-only task, participants reported their perception of the facial expression (rather than the body posture) in the face-in-context task as instructed.

Both the autistic and the non-autistic groups showed a clear influence of body posture on facial expression perception, as indicated by a significantly larger PSE for facial expressions displayed on an angry body (median Autistic: 0.759; Non-Autistic: 0.672) compared to a disgusted body (Autistic: 0.2932; Non-Autistic: 0.362) (Fig. 2C; Autistic: Wilcoxon signed rank test z = 5.625, *p* < 0.001; Non-Autistic: z = 6.308, *p* < 0.001). Importantly, the PSE difference between facial expressions presented on a fully angry body vs. on a fully disgusted body in the autistic group (median: 0.625) was significantly higher than the PSE difference in the non-autistic group (median: 0.343) (Wilcoxon rank sum test z = 1.982, *p* = 0.048), indicating a greater influence of body posture on facial expression perception in the autistic group relative to the non-autistic group.

**Fig. 3:**
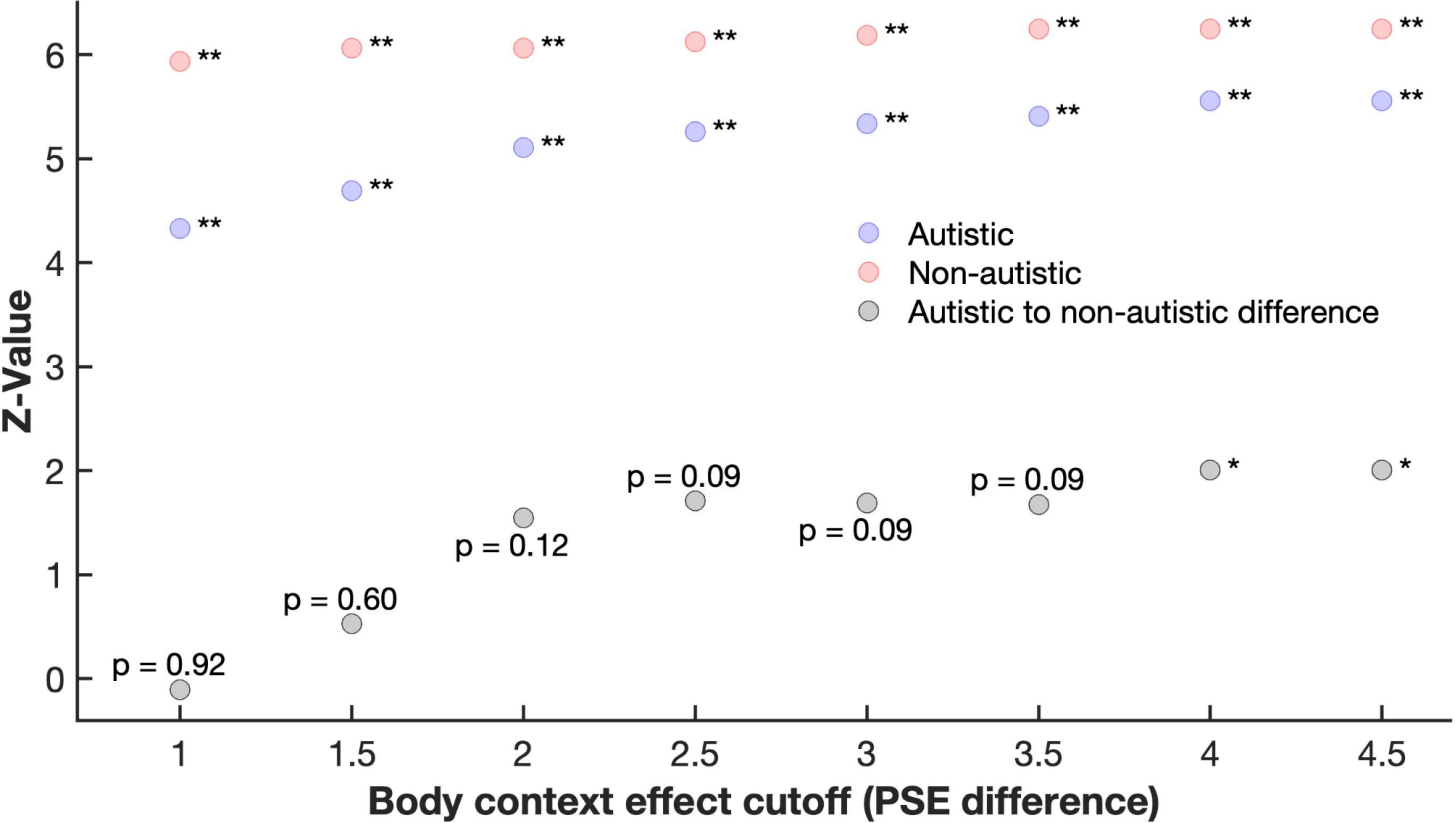
Robustness analysis of the body context effect in the autistic and non-autistic sample as a function of increasing PSE difference cut-offs/exclusion criteria. The coloured points indicate the z-values of the Wilcoxon Signed Rank test for the autistic (blue) and non-autistic (red) groups, assessing the difference in PSE between the conditions in which face morphs were shown either in a disgusted or angry body context. The grey points indicate the z-values of the Wilcoxon Sum Rank test, assessing whether there is a difference in the body context effect between groups. Note: each Wilcoxon Rank Sum test is based on different sample sizes, which are determined by the number of participants at or below the body context effect cutoff (PSE difference) shown on the x-axis. * *p* < 0.05, ** *p* < 0.01.

Given that some individuals showed large differences in PSEs – our measure of the body context effect – we conducted an analysis to assess the robustness of our findings against exclusion of participants with extreme PSE difference values (Fig. 3). This analysis showed that the body context effect per se was very robust in both groups, with the pattern of findings not depending on specific exclusion criteria. The findings were less clear for the difference between groups in the body context effect (Fig. 3, grey points): with less extreme exclusion criteria – i.e., when many participants with larger PSE differences were retained – the findings largely mirrored the pattern found for the whole sample. However, with more extreme exclusion criteria – i.e., when fewer participants with large PSE differences were retained – no difference between the groups emerged. This finding suggests that the difference in the body context effect observed in the full sample between autistic and non-autistic individuals was driven by a difference in the number of individuals with a very strong influence of body context on facial expression perception.

### Relationship between facial expression and body posture perception and the body context effect

**Fig. 4:**
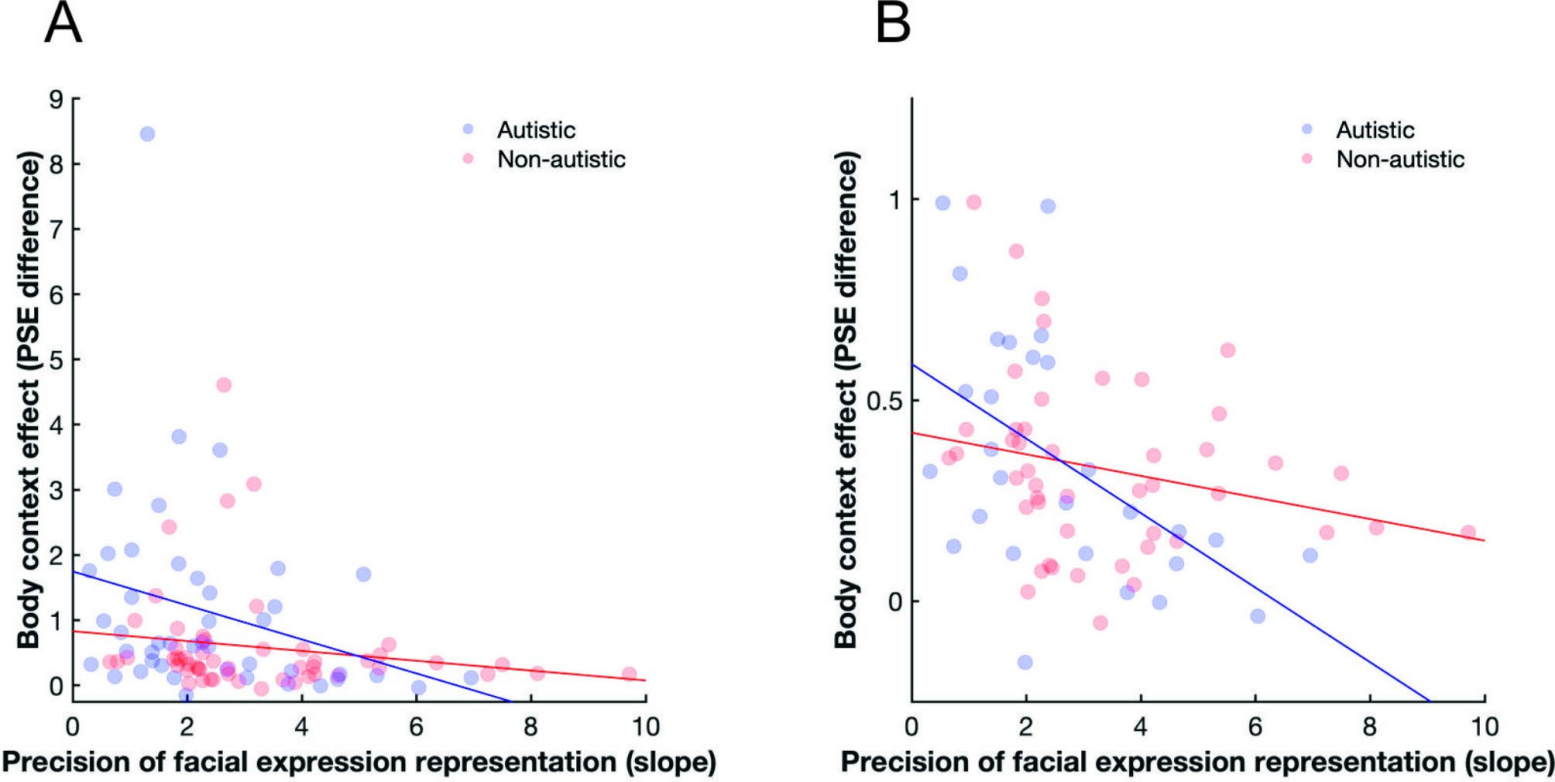
Relationship between the body context effect (as indexed by the change in PSE) and the precision of facial expression representations (as measured by the slope of the psychometric function in the isolated facial expression task). (**A**) Relationships with all participants included, and (**B**) only participants with a PSE difference smaller than 1 included (see robustness analysis depicted in Fig. 5).

The influence of body posture on facial expression perception (as indexed by the PSE difference) had a negative relationship with the precision of facial expression representations (as indexed by the slope of psychometric function in the isolated facial expression task) in both the autistic sample (Fig. 4A; Spearman’s rho = -0.39, *p* = 0.0094) and the non-autistic sample (Fig. 4A; rho = -0.30, *p* = 0.028). There was no significant relationship between body posture discrimination ability and the influence of body posture on facial expression categorisation in the autistic (rho = -0.17, *p* = 0.26) and the non-autistic groups (rho = -0.23, *p* = 0.1).

**Fig. 5:**
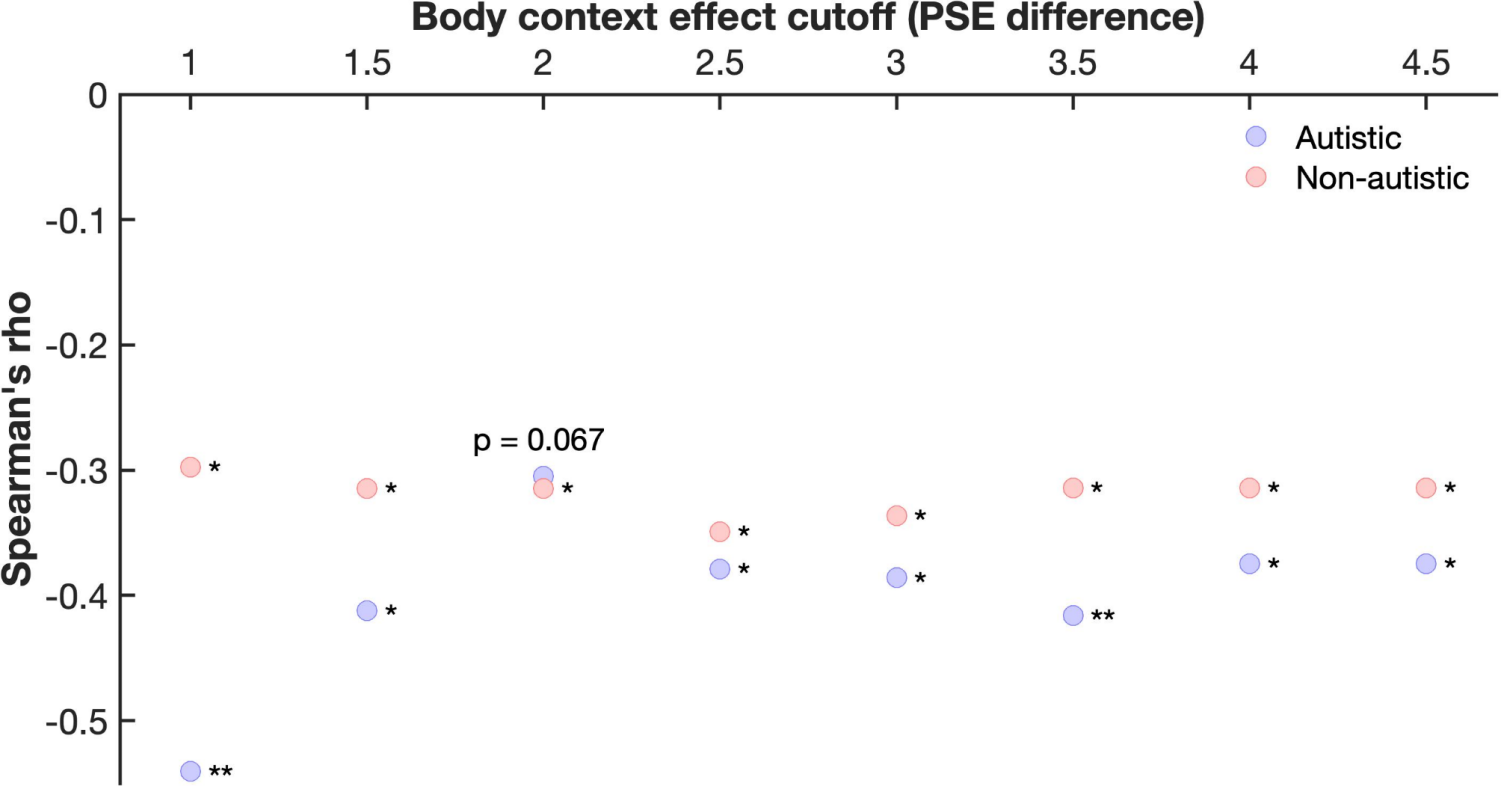
Robustness analysis of the relationship between precision of facial expression representations and body context effect. All except for one of the calculated correlations support the conclusion of a significant negative relationship in both groups. Note: each correlation coefficient (Spearman’s rho) is based on different sample sizes, which are determined by the number of participants at or below the body context effect cutoff (PSE difference) shown on the x-axis. * *p* < 0.05, ** *p* < 0.01.

As illustrated in Fig. 4A, several participants had a very large difference in their PSE values. We therefore conducted a robustness analysis, in which we used different cut-offs for the PSE difference above which we excluded participants (Figs. 4B and 5). All except one of the calculated correlations, which showed a trend result, indicated a significant negative relationship between precision in facial expression representation and body context effect in both groups, suggesting an effect that is robust against potential outliers.

## Discussion

The current study investigated the influence of body posture on facial expression perception in autistic and non-autistic adults. We found that body posture had a significant influence on facial expression perception in both groups, but autistic individuals showed a *greater* influence of body posture compared with non-autistic individuals. We also found reduced precision of isolated facial expression representations, but no difference in the precision of isolated body posture representations, in autistic individuals compared with non-autistic individuals. Within each group, the precision of isolated facial expression representations was related to the body context effect, with lower precision leading to an increased influence of body posture. Taken together, our results show that perception of facial expressions in individuals with Autism is more strongly influenced by body posture compared to non-autistic individuals, and that a reduced precision in facial expression representations is likely a key factor driving this effect.

Our finding of a significant influence of body posture on facial expression categorisation in non-autistic adults, as well as in autistic adults, is consistent with the findings of previous research^2–4,17^. However, the comparison between the autistic and non-autistic groups differed with the only previous study on this topic^17^. Contrasting with the current work, Brewer and colleagues^17^ found no difference in the influence of body context between the autistic group and the non-autistic group, as well as no difference in the precision of facial expression representations between the groups. Importantly, however, from a Bayesian perspective both sets of results are consistent. Specifically, a Bayesian account would predict that the biasing influence of body context is related to the reliability or precision with which isolated facial expression cues are represented. Our data indicate that this prediction holds for both the autistic and non-autistic groups. In both groups, the body context effect is larger in individuals with less reliable facial expression representations compared to those with more reliable representations. The difference in the body context effect *between* the groups might thus be a consequence of the difference in the reliability of facial expression representations: as predicted, the autistic group showed less reliable representations than the non-autistic group and might therefore be more strongly influenced by body context. In the study by Brewer and colleagues^17^, the two groups did *not* differ in how reliably they represented facial expressions. The fact that these authors did not find a difference in the body context effect between groups is thus expected from a Bayesian perspective. In summary, the contrasting results between our work and the only previous study focussing on the influence of body context on facial expression perception in autism are both consistent with a Bayesian interpretation of the body context effect.

Research on facial expression recognition in autistic individuals has produced conflicting results, with some studies finding difficulties in autistic compared to non-autistic individuals and others finding no differences (for reviews, see:^13,15^). One potential explanation for these mixed findings is the genuine heterogeneity of facial expression perception in autism, with different subgroups showing different extents of difficulties in facial expression perception^18^. Our autistic sample was more than twice the size of the autistic sample in Brewer and colleagues^17^, increasing the probability of including individuals from subgroups with pronounced difficulties in facial expression perception. Additionally, it is worth noting that most data exclusions in our study were due to chance responding in the tasks involving facial expression discrimination (face-only and face-in-context tasks), and that there were almost 80% more data exclusions in the autistic group than in the non-autistic group. Such chance responding for facial expressions, while body posture discrimination was unaffected, is a further reflection of the difficulties in facial expression discrimination in our autistic participants, and suggests that the group differences reported here in both the precision of facial expression representations and the influence of body posture on facial expression perception are underestimated.

Another potential factor contributing to the inconsistencies in the literature may be the type of facial expression stimuli used. Studies that rely on stereotypical 100% facial expressions may not be sensitive enough to detect subtle differences between autistic and neurotypical groups due to ceiling effects associated with using these stimuli^13^. In our study, the use of facial expression morphs allowed us to characterise facial expression recognition in a more sensitive manner. The reduced precision of facial expression representations that we observed in autistic individuals is consistent with the results of a previous study by Wang and Adolphs^12^, who also used facial expression morphs and found a similarly reduced precision of facial expression representations in autistic individuals.

By contrast, our results suggest that discrimination of emotion in body postures is relatively unaffected in autistic individuals, with both autistic and non-autistic individuals showing comparable discrimination of body posture. It should be noted, however, that our study was a forced-choice paradigm with a choice between two body emotions, and the body postures of anger and disgust were generally less confusable (i.e. associated with greater precision of the underlying representations) than facial expressions of anger and disgust. Nevertheless, the results are consistent with previous research reporting no differences in recognition accuracy of body posture stimuli in autistic compared to non-autistic individuals^16^.

As mentioned above, in both groups, we found a relationship between the precision of facial expression representations and the influence of body posture. This link between facial expression processing and body context effect has also been found in children and adolescents^7^, with younger children showing reduced precision in facial expression representations and a greater reliance on body posture in making judgments of facial expression, and vice versa for older children. This consistent pattern of findings across typical development^7^, non-autistic adults, and autistic adults provides support for the notion that shared mechanisms drive the integration of emotion information from face and body across these groups. The extent to which such principles apply more widely to the integration of other social signals from face and body, or indeed other contextual signals, remains an open and interesting question.

With respect to the difference in body context effects between the two groups, it is important to note that this finding cannot be explained by avoidance of the face and/or eye region in the autistic group and judgments based purely on body context. Avoidance of eye contact is a key diagnostic feature of Autism^19^. However, the high consistency in performance of autistic individuals across the isolated facial expression and face-in-context tasks suggests that autistic individuals were discriminating facial expressions in the face-in-context task, rather than focussing on the body posture only. Additionally, based on our exclusion criteria, any participants, who simply responded according to body posture, would have been removed from the analysis due to an inability to fit psychometric functions to the data.

One limitation of our study is the fact that the body postures have not been validated in the way that the facial expressions have been. When we created the body posture stimuli however, we piloted their recognisability. Participants had no difficulty identifying the emotion conveyed by the body posture. Importantly, based on our data here of the categorisation of isolated body postures, participants perform better in identifying isolated body postures than isolated facial expressions (steeper slopes of psychometric functions for isolated bodies than for isolated faces). Furthermore, the body postures have been used in previously published research^7^.

A further limitation of our study is the focus on the emotions of anger and disgust, and male stimuli only. The emotions of anger and disgust were chosen due to a robust effect of body context compared to other emotions^2^, and to be consistent with Brewer et al.^17^. A robust influence of body posture was important for maximising sensitivity to individual differences within and between our two groups of observers. Furthermore, disgust and anger can be conveyed by simple, clearly identifiable body postures. By contrast, other postures like sadness and happiness are less easily conveyed clearly via a simple posture^20^. Our use of male stimuli only was to ensure perceptual similarity, as male facial expressions of anger are typically recognised more quickly and accurately than female facial expressions of anger^21^. Future research should include other emotions, as well as female faces and bodies, to further investigate principles underpinning integration. However, there is no reason to believe that the integration of other emotions, and female faces/bodies, would not be governed by similar underlying principles. Indeed, previous research suggests that the bias towards other body emotions is smaller but not absent^2^. Several researchers have studied congruency effects with a broader range of emotions in the face and body, with findings suggesting that body posture influences facial expression perception across emotions, albeit to varying degrees^22,23^. Thus, while the principles underlying integration may be similar, broadening the scope to other emotions may raise interesting questions regarding additional factors which may influence integration. For instance, research has suggested that sensorimotor information more strongly characterises some body postures (e.g., anger) than others (e.g., sadness)^24–26^, which may influence integration with facial expression information.

In summary, our results demonstrate both similarities and differences between autistic and non-autistic individuals regarding the perception of facial expressions, body postures and facial expressions in the context of a body posture. A significant reduction was found in the precision of facial expression representations in autistic individuals, relative to non-autistic individuals, but there was no difference in the precision of body posture representations between groups. Autistic individuals also showed a greater reliance on body posture in making their judgements of facial expression than non-autistic individuals. Across each group, the influence of body posture on facial expression perception was related to the precision of facial expression representations. Our results highlight that similar principles govern the integration of facial expression and body posture information in autistic and non-autistic individuals.

## Methods

### Participants

A power analysis using G*Power 3.1^27^ revealed that, to achieve 80% power, sample sizes of 37–39 participants were needed in each group (depending on whether the data is normally or non-normally distributed) to detect a difference between groups in the influence of body context on facial expression perception. Because the study was being conducted online and these psychophysical tasks lead to the exclusion of many participants even when testing in person^7^, we aimed for sample sizes of ~ 75 in each group to ensure we would retain a large enough sample.

A total of 78 autistic adults and 72 non-autistic adults were recruited through Prolific (Prolific Team, 2019). The autistic individuals, as well as the matched non-autistic individuals in this study were drawn from larger samples used in previous online autism research conducted using the Prolific autism screener (e.g^28^). Specifically, autistic participants in the sample underwent multiple verification processes; they were all UK residents and had received a clinical diagnosis of ASD according to DSM or ICD criteria^19,29^. The diagnosis was received from a healthcare professional in a formal clinical setting, such as a hospital or specialist autism service, when the participant was either a child or an adult. In line with previous studies that have recruited large samples of autistic people^30–32^, participants provided detailed information about their diagnosis (e.g. ASD, Asperger syndrome), their clinician (e.g., Psychiatrist, Psychologist), and the diagnosis location. These details were also confirmed by the participant during the screening process before participation in the study. Individuals who self-identified as autistic or those seeking or awaiting diagnosis were not eligible to participate. Many participants had recently participated in other autism research studies^33^, including in-person^34^. The 16-item International Cognitive Ability Resource (ICAR^35^) was administered to all autistic and non-autistic participants as the measure of general cognitive ability to ensure that the groups did not significantly differ on this task. This well-validated and psychometrically robust measure is designed for online studies, and includes matrix reasoning, 3D rotation, verbal reasoning, and letter and number series. Scores range from 0 to 16, with higher scores indexing greater cognitive ability. The ICAR for the current sample was collected as part of a previous online study (for further details, see^36^).

Following data exclusions outlined below, the final autistic (*N* = 44) and non-autistic groups (*N* = 53) in the current study were closely matched on age (autistic age M = 30.7 ± 9.3 years, range 19–58; non-autistic age M = 33.6 ± 10.5 years, range 19–58; W = 1189.5, *p* = 0.251), cognitive ability (autistic score M = 9.0 ± 3.5; non-autistic score M = 9.5 ± 3.2; t(99) = -0.673, *p* = 0.503), and gender (autistic group = 13 males; non-autistic group = 24 males;  $\chi$^2^(1, *N* = 97) = 2.52, *p* = 0.11).

This study was approved by Cardiff University School of Psychology Ethics Committee. The study adhered to the ethical standards of the Declaration of Helsinki. Participants provided informed consent via an online Qualtrics form (Qualtrics, Provo, UT). Participants were paid for their participation through Prolific.

### Stimuli

Male facial expressions of anger and disgust were selected from the Karolinska Directed Emotional Faces database^37^ and Radboud faces database^38^. Four Caucasian male faces were selected and their angry and disgusted facial expressions were morphed together for each of the four identities using FantaMorph software (FantaMorph Pro, Version5). This created a morph continuum between angry and disgusted facial expressions for each identity. The facial expression morphs in this morph continuum changed in increments of 10% (10–90%), resulting in 9 facial expression morphs between anger and disgust for each identity.

**Fig. 6:**
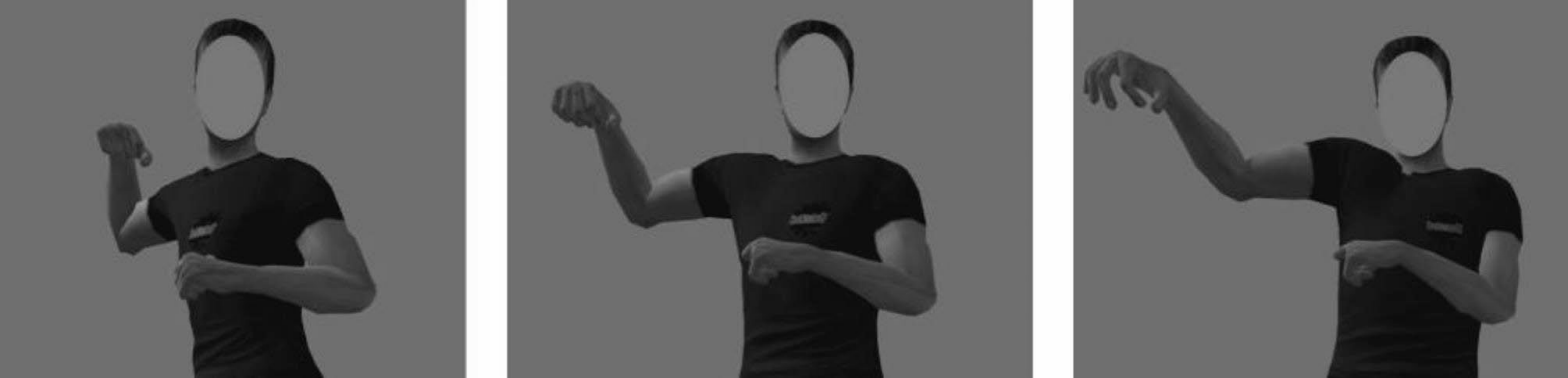
Example body posture stimuli for one identity. From left to right: 100% angry body posture, 50% anger/50% disgust body posture, 100% disgust body posture. Covered facial expression stimuli from Karolinska Directed Emotional Faces database^37^.

To create the body posture morph stimuli, a motion capture suit and a Unity 3D game engine was used^39^. A male adult actor was instructed to pose in certain body postures expressing anger and disgust (based on the poses used in^2^), whilst wearing a motion capture suit with motion trackers distributed over the whole body^40^. The Unity 3D game engine was then used to visualise the body postures captured by the actor in the motion capture suit. Four unique body postures were produced by the actor displaying slightly different poses, the body composition and clothing given to each of these unique postures were also different – resulting in four “identities”. Weighted averages of these angry and disgusted body postures were then used to produce body posture morphs between anger and disgust for each of the four identities. As with the facial expression morphs, the body posture morphs changed in increments of 10% (10–90%), resulting in 9 morph levels per identity (Fig. 6). The body posture morphs described here have been used in previous research^7^.

For the face-in-context task, the face and body stimuli were then combined (GNU Image Manipulation Program, Version 2.10) to create whole-person stimuli for each separate identity. Specifically, the 100% body postures (either 100% angry or 100% disgusted) were combined with each facial expression morph. For each identity, there were thus 9 facial expression morphs shown on a fully angry body posture, and 9 facial expression morphs shown on a fully disgusted body posture.

### Design

Stimuli were presented using PsychoPy^41^, hosted on Pavlovia (www.pavlovia.org). Participants accessed the task via an online link. Participants could only take part in the study if they were accessing the link via a desktop computer or laptop, to keep the presentation of stimuli as consistent as possible.

There were three tasks (a face-only, a body-only, and a face-in-context task), which were presented in a randomised order for each participant. For each trial, participants were presented with a stimulus and asked to judge whether the stimulus was disgusted or angry. Each unique stimulus was repeated 3 times in each task, such that there were 12 repetitions per morph level (4 identities x 3 repeats). There was a total of 108 trials in the face-only and body-only tasks, and 216 in the face-in-context task (108 trials each for the facial expression morphs on a fully angry or a fully disgusted body posture). Stimulus presentation was pseudorandomised for each task to ensure that the same identity was never shown in two consecutive trials. Before each task, there was a practice session consisting of 10 trials to ensure the participant knew what was expected of them. Stimuli in the practice sessions were a random subset of the stimuli used in the actual tasks.

**Fig. 7:**
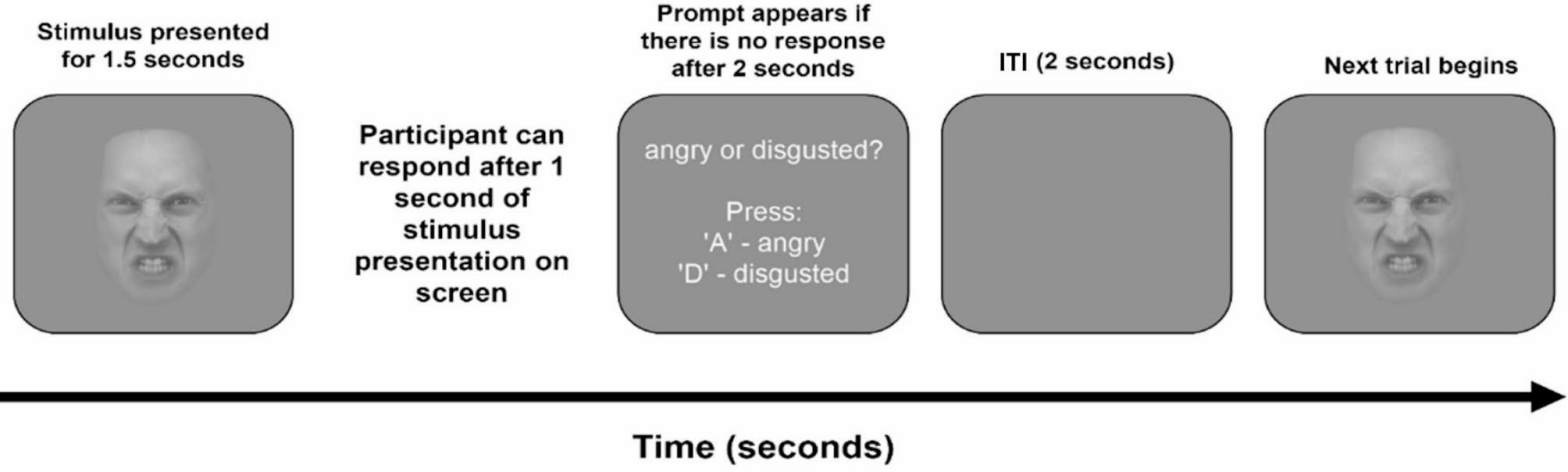
Example trial. Example stimulus is from isolated facial expression task, but trial structure was identical for all tasks. Facial expression stimulus from Karolinska Directed Emotional Faces database^37^. ITI Intertrial Interval.

In each trial (Fig. 7), a stimulus was presented for 1.5 s on a grey background, and participants could respond after the stimulus had been on the screen for 1 s. For the face-only and body-only tasks, participants were asked to categorise the facial expression and body posture respectively, as being either angry (keypress ‘A’) or disgusted (keypress ‘D’). In the face-in-context task, participants were instructed to ignore the body posture and judge whether the facial expression was angry or disgusted. A prompt would appear if no response was made after 2 s, reminding participants of the response options. Progression to the next trial only occurred once the participant had responded. There was an intertrial interval of 2 s. The duration of the face-only and body-only tasks were ~ 7 min each, and the face-in-context task was ~ 13 min. Thus, the total duration of the study was approximately 30 min.

Psychometric functions based on a cumulative Gaussian were fitted to estimate each observer’s point of subjective equality (PSE) and precision of representation (indexed by the slope parameter) for each task, using MATLAB R2019b and existing functions within the Palamedes toolbox^42^. Lapse rate was treated as a fixed parameter, set to 0.03. The slope values for the face-only and body-only tasks provided estimates of the precision of the facial expression and body posture representations respectively, whereas the difference between the PSEs for the two fitted psychometric functions in the face-in-context task provided an estimate of the influence of body context on facial expression perception (the difference in ‘% disgust in the facial expression stimulus’ for the same probability of perceptual judgment to occur, regardless of body posture). For our analysis, we excluded participants who were responding at chance level and/or who responded identically across all morph levels in any of the 3 tasks. For the autistic group, this resulted in 34 participant exclusions, while for the non-autistic group this resulted in 19 participant exclusions. The higher number of exclusions in the autism group is in keeping with their greater difficulty in discriminating facial expressions, as most exclusions were related to the tasks involving discrimination of facial expressions (face-only and face-in-context tasks), whilst performance on the body-only task was similar to the non-autistic group.

Statistical analyses were carried out with the software R (The R Foundation for Statistical Computing). Data were non-normally distributed, so non-parametric tests were used throughout.

## Data Availability

The data generated and analysed during the current study are available from the corresponding author on reasonable request.
